# Blended alginate/collagen hydrogels promote neurogenesis and neuronal maturation

**DOI:** 10.1016/j.msec.2019.109904

**Published:** 2019-11

**Authors:** Samuel R. Moxon, Nicola J. Corbett, Kate Fisher, Geoffrey Potjewyd, Marco Domingos, Nigel M. Hooper

**Affiliations:** a Division of Neuroscience and Experimental Psychology, School of Biological Sciences, Faculty of Biology, Medicine and Health, The University of Manchester, Manchester Academic Health Science Centre, Manchester M13 9PL, UK; b School of Mechanical, Aerospace and Civil Engineering, Faculty of Science and Engineering, The University of Manchester, Manchester M13 9PL, UK

**Keywords:** Alginate, Collagen, iPSC, Neuron, Neurogenesis, 3D tissue models

## Abstract

Brain extracellular matrix (ECM) is complex, heterogeneous and often poorly replicated in traditional 2D cell culture systems. The development of more physiologically relevant 3D cell models capable of emulating the native ECM is of paramount importance for the study of human induced pluripotent stem cell (iPSC)-derived neurons. Due to its structural similarity with hyaluronic acid, a primary component of brain ECM, alginate is a potential biomaterial for 3D cell culture systems. However, a lack of cell adhesion motifs within the chemical structure of alginate has limited its application in neural culture systems. This study presents a simple and accessible method of incorporating collagen fibrils into an alginate hydrogel by physical mixing and controlled gelation under physiological conditions and tests the hypothesis that such a substrate could influence the behaviour of human neurons in 3D culture. Regulation of the gelation process enabled the penetration of collagen fibrils throughout the hydrogel structure as demonstrated by transmission electron microscopy. Encapsulated human iPSC-derived neurons adhered to the blended hydrogel as evidenced by the increased expression of α1, α2 and β1 integrins. Furthermore, immunofluorescence microscopy revealed that encapsulated neurons formed complex neural networks and matured into branched neurons expressing synaptophysin, a key protein involved in neurotransmission, along the neurites. Mechanical tuning of the hydrogel stiffness by modulation of the alginate ionic crosslinker concentration also influenced neuron-specific gene expression. In conclusion, we have shown that by tuning the physicochemical properties of the alginate/collagen blend it is possible to create different ECM-like microenvironments where complex mechanisms underpinning the growth and development of human neurons can be simulated and systematically investigated.

## Highlights

• Alginate and collagen are blended to create a bespoke hydrogel that mimics aspects of brain ECM.
• Encapsulated human pluripotent stem cell derived neurons adhere to the hydrogel matrix and form 3D neural networks.
• Neuronal differentiation and maturation is promoted within the hydrogel matrix.
• Mechanical properties of the hydrogel can be easily tuned to optimise neurogenesis.
• The hydrogel presents a platform for studying neuronal function and dysfunction in health and disease.

## Graphical abstract

**Figure:**
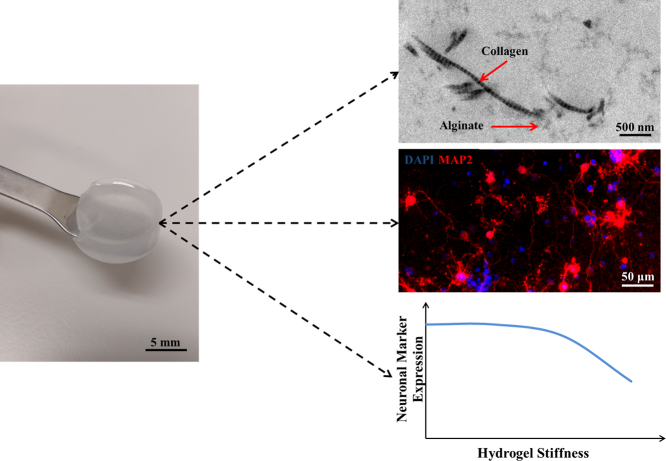

## 1 Introduction

Brain extracellular matrix (ECM) is unique in structure due to a high content of linear polysaccharides when compared to other tissues [1]. In addition, brain ECM is often referred to as a soft matrix with an elastic modulus reported to be ~0.1–1.5 kPa [2,3]; orders of magnitude lower than tissues such as bone, cartilage and muscle [[4], [5], [6]]. Structurally, brain ECM is a heterogeneous network of polysaccharide-protein complexes called proteoglycans, the most abundant of which are formed by interactions involving hyaluronic acid (HA), a linear, carboxylated polysaccharide that associates with multiple proteins [7]. A key site of HA-protein interaction is at the carboxylic acid functional group present on the glucuronic acid residues of the HA polysaccharide chain. Numerous proteins, including versican and aggrecan, have been reported to ionically bind to HA at this residue and form proteoglycan complexes [8]. Proteoglycans are critical to brain function and have been shown to regulate the adhesion of neurons, subsequent neurite outgrowth and the formation of complex neural networks [9,10].

In vitro studies of the brain, however, often lack replication of the complex environment within which neural cells develop and function in both health and disease [11]. 2D monolayer cell cultures are still commonly utilised and, while surface coatings can be used to introduce brain ECM components, the complex 3D network of neural cells and matrix found in vivo cannot be recapitulated [12]. Applying biomaterial systems to replicate aspects of the brain ECM for in vitro studies of neurogenesis and neurodegeneration has consequently gained significant interest in recent years [13,14]. Direct use of brain ECM components is, however, difficult due to low bioavailability and high cost. Additionally, substrates that comprise brain ECM can be difficult to incorporate into a 3D cell culture environment. Hyaluronic acid, for example, requires chemical modification in order to allow for crosslinking into a hydrogel for cell culture applications. This process involves use of potentially cytotoxic concentrations of chemical crosslinkers or application of gelation conditions that can compromise cell viability such as high temperatures, alkaline pH and UV exposure [[15], [16], [17]]. Consequently, studies often utilise hydrogels comprised of naturally occurring polysaccharides or polypeptides that support neural cell culture by recapitulating some of the structural and mechanical elements of brain ECM as opposed to direct use of brain ECM components [[18], [19], [20]]. Such components often offer low cost and exhibit gelation mechanisms that are more suitable for maintaining cell viability [21].

Alginate is a linear polysaccharide with an abundance of carboxylated guluronic and mannuronic acid residues that has structural similarities to hyaluronic acid [22]. The polymer chain carries a net negative charge which allows for solutions of alginate to be covalently crosslinked into polysaccharide hydrogels via interactions between divalent cations and the guluronic acid residues [23]. Gelation occurs rapidly and can be initiated under physiological conditions, allowing for maintenance of cell viability during the encapsulation process [24]. The mechanical properties of alginate hydrogels can be easily tuned by varying either the polymer or crosslinker concentration. Consequently, alginate hydrogels can be manipulated to reflect the matrix stiffness of the brain [25]. However, a lack of cell adhesion motifs on the polysaccharide chain means alginate has to be either chemically modified or blended with cell-adhesive materials in order to promote neural development [[26], [27], [28], [29]].

Collagen has previously been proposed as a material that can be combined with alginate to create a hydrogel that is mechanically tuneable and allows for cellular adhesion [30]. Collagen hydrogels often exhibit low matrix stiffness and contain integrin binding sites for cell-matrix interactions [[31], [32], [33], [34]]. Although alginate/collagen blended hydrogels have been used to reproduce connective, renal and ocular tissues [[35], [36], [37]], they have yet to be explored as substrates for the culture of neural cells. In this study, blended hydrogel networks of alginate and collagen have been developed as accessible platforms for the 3D culture of induced pluripotent stem cell (iPSC) derived neurons and to test the hypothesis that the physical and structural properties of the hydrogel influence the growth and development of human neurons. The two materials were physically mixed into a pre-gelled solution and reticulated under conditions that allowed for formation of a polysaccharide network penetrated by collagen fibrils without compromising cell viability. Encapsulated neurons adhered to the resulting hydrogel matrix via integrin binding sites within the collagen fibrils and formed neural processes. In addition, the 3D hydrogel network promoted key processes of neuronal maturation and synapse formation, namely, translocation of pre-synaptic transport proteins from the cell soma down the neurites and development of increased neurite branching. The physical and structural properties of the hydrogel were easily tuned, providing encapsulated cells with extracellular cues that optimised expression of neurogenesis and synaptogenesis markers.

## 2 Materials and methods

### 2.1 Materials

Sodium alginate (~44% G content) and collagen (PureCol® EZ Gel) were purchased from Sigma Aldrich (UK). The OX1-19 iPSC line was generated and provided by S. Cowley at the University of Oxford (UK) [38]. Neurobasal media, N2 and B27 supplements were purchased from Life Technologies™ (UK). Primary and secondary antibodies for immunofluorescence microscopy were purchased from Abcam (UK). RNA isolation, cDNA synthesis and primer kits for RT-PCR were purchased from Qiagen, Bio-Rad and Primerdesign (all UK), respectively. All other reagents were purchased from Sigma Aldrich (UK) and used without further modification.

### 2.2 Cortical neuron differentiation of human iPSCs

Cortical neurons were generated from iPSCs using a previously published protocol [39]. Briefly, undifferentiated cells were maintained at 37 °C and 5% CO_2_ on Matrigel coated tissue culture plastic (TCP) in mTeSR1 media supplemented with 50 U/ml penicillin and 50 μg/ml streptomycin. Dual SMAD signalling inhibition with 1 μM dorsomorphin and 10 μM SB431452 was performed to induce neural differentiation [40]. During differentiation, immunofluorescence microscopy was used (as in 2.7) to analyse cell phenotype and morphology. At 35 days post-induction, the resulting neural progenitor cells were segregated for encapsulation in alginate/collagen hydrogels. Neural progenitors (NPCs) were then cultured using neural maintenance media (NMM) with media changes every 2–3 days.

### 2.3 Hydrogel formation and cell encapsulation

**Fig. 1:**
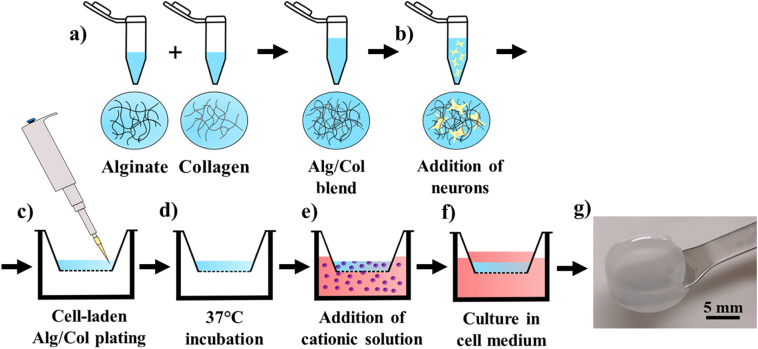
Schematic demonstrating the fabrication process of cell-laden alginate/collagen hydrogels. a) physical mixing of alginate and collagen, b) incorporation of neurons, c) plating of pre-gelled solution, d) incubation at 37 °C to trigger collagen fibrillogenesis, e) cationic crosslinking of alginate, and f) subsequent culture of neuron-laden alginate/collagen hydrogels, g) photograph of the resulting hydrogel sample.

Alginate solutions (40 mg/ml) were created by dissolving alginate powder in de-ionised water under constant stirring. Solutions of 20 mg/ml alginate were subsequently generated by mixing 40 mg/ml alginate with NMM at a 1:1 volumetric ratio. Collagen (PureCol® EZ Gel, 5 mg/ml) was mixed with alginate in a 1:1 ratio to create a final pre-gel solution of 10 mg/ml alginate and 2.5 mg/ml collagen. To create cell-laden hydrogels, day 35 NPCs were mixed with alginate-collagen solutions at a seeding density of 1 × 10^6^ cells/ml. The solution was pipetted into 12-well cell culture inserts (0.5 mm pore diameter) and incubated at 37 °C for 4 h to trigger collagen fibril formation. Hydrogels were then treated with CaCl_2_ (150 mM unless otherwise stated) for 10 min to induce ionotropic alginate gelation (Fig. 1). The resulting gels were then washed with phosphate-buffered saline (PBS; 0.9 mM CaCl_2_, 0.5 mM MgCl_2_, 2.7 mM KCl, 1.5 mM KH_2_PO_4_, 138 mM NaCl, 8 mM Na_2_HPO_4_) and cultured in NMM at 37 °C and 5% CO_2_.

### 2.4 Gelation kinetics

Oscillatory rheology was applied to pre-gelled hydrogel solutions using a Discovery HR-2 rheometer (TA Instruments, UK) equipped with a 20 mm parallel plate geometry. Gelation kinetics were analysed by monitoring storage modulus (G′) and loss modulus (G″) over 60 min at 37 °C. Strain and oscillatory frequency were kept constant at 0.5% and 10 rad/s respectively. After holding for 40 min to allow for collagen fibrillation, 150 mM CaCl_2_ was introduced to trigger ionotropic gelation of alginate solution. This process was also conducted on control samples of 5 mg/ml collagen and 20 mg/ml alginate separately to obtain gelation profiles of the raw materials.

### 2.5 Microstructural characterisation of blended hydrogels

Cell-laden alginate/collagen hydrogels were fixed with 4% formaldehyde and 2.5% glutaraldehyde in 0.1 M Hepes buffer (pH 7.2). Samples were then post-fixed with 1% osmium tetroxide and 1.5% potassium ferrocyanide in 0.1 M cacodylate buffer (pH 7.2) for 1 h and in 1% uranyl acetate in water overnight. The fixed cell-laden hydrogels were then dehydrated in ethanol, infiltrated with TAAB's Low Viscosity resin and polymerized for 24 h at 60 °C. Sections of the resulting hydrogels were cut with a Reichert Ultracut ultramicrotome (Leica Microsystems, UK) and observed with a FEI Tecnai 12 Biotwin microscope (FEI™ Company, USA) at 100 kV accelerating voltage. Images were taken with a Gatan Orius SC1000 CCD camera (Gatan, UK). Additionally, alginate/collagen hydrogels were analysed with bright field confocal microscopy with 20 mg/ml alginate hydrogels used as a control. Samples were fixed for 20 min with 4% paraformaldehyde (PFA) before being washed with PBS containing 50 mM NH_4_Cl to inactivate excess fixative. The hydrogels were subsequently washed with PBS and imaged using a Leica TCS confocal microscope (Leica Biosystems, UK).

### 2.6 Integrin expression

Prior to performing quantitative PCR, RNA was isolated from cell-laden hydrogels using an RNeasy RNA isolation kit. Complementary DNA (cDNA) was subsequently generated using an iScript cDNA synthesis kit and incorporated into a 20 μl qPCR reaction mix with Precision OneStepPLUS SYBR Green Dye and Primerdesign custom PCR primers. Glyceraldehyde 3-phosphate dehydrogenase (GAPDH) was used as a housekeeping gene. Expression of collagen-binding integrin subunits α1, α2 and β1 was analysed to probe for cell-matrix interactions. Relative expression was subsequently calculated using the double delta CT quantification method [41] with cells cultured in 20 mg/ml alginate (no cell adhesion domains) used as a control.

### 2.7 Cell morphology analysis

Cell-laden hydrogels were removed from 3D culture at day 45 post-neural induction (10 days post-encapsulation), fixed with 4% PFA for 20 min and subsequently washed for 10 min with PBS containing 50 mM NH_4_Cl. Cells were permeabilised for 15 min in PBS with 0.2% Triton X-100, washed with PBS and blocked for 2 h in PBS with 10% donkey serum. Cell cultures were subsequently incubated with primary antibody for human βIII-tubulin (1:500 in 10% donkey serum) overnight at 4 °C. Samples were then washed with PBS, incubated for 1 h with fluorescently-conjugated secondary antibody (1:500 in 10% donkey serum) and DAPI (1:500 in PBS) before being washed with PBS and 0.2% Tween-20. Fluorescently-labelled cultures were then embedded in 6% agarose and sliced into 200 μm sections using a Leica VT1000S vibratome (Leica, UK) to address issues commonly reported in imaging cell-laden hydrogels in bulk such as light refraction, optical turbidity and restrictions on magnification [[42], [43], [44]]. High resolution fluorescence microscopy using an EVOS FL Cell Imaging System (ThermoFisher, UK) was applied to image encapsulated cells at 10×, 20× and 100× magnification. Sections were prepared across the entire z axis of the hydrogels and images were taken from every sample.

### 2.8 Quantification of synaptophysin density

Cell-laden hydrogels were prepared for immunofluorescence microscopy (IFM) as in 2.7 using primary antibodies for human microtubule associated protein 2 (MAP2) and synaptophysin (1:500 and 1:250, respectively, in 10% donkey serum) at 25 and 55 days post-encapsulation (60 and 90 days of neural induction). Once processed and sliced, the samples were imaged at 100× magnification using oil. ImageJ was subsequently used to determine the fluorescent density of synaptophysin in the neurites of encapsulated cells with images corrected for removal of background fluorescent signal.

### 2.9 Neuronal branch point analysis

Hydrogels were prepared for IFM as in 2.7 and 2.8 using primary antibody for human MAP2 at 25 and 55 days post-encapsulation (60 and 90 days of neural induction). ImageJ was used to analyse the number of branch points along neurites. Data was standardised to represent the average number of branch points per 50 μm of neurite.

### 2.10 Mechanical tuneability

The mechanical tuneability of the alginate/collagen hydrogel was demonstrated using frequency sweeps. Hydrogels were reticulated with 3 concentrations of CaCl_2_ (75, 150 and 300 mM) prior to loading onto the rheometer. Storage and loss moduli were subsequently determined in response to increasing oscillatory frequency (1–10 rad/s) with a constant strain and temperature of 0.5% and 37 °C, respectively.

### 2.11 Hydrogel porosity and permeability

The effect of CaCl_2_ concentration on the porosity of alginate/collagen hydrogels was determined by solvent replacement. Hydrogels were crosslinked with 75, 150 and 300 mM CaCl_2_, freeze dried for 24 h and weighed. Samples were then soaked in absolute ethanol for 24 h and re-weighed after excess ethanol was removed by blotting. Porosity (%) was then calculated using Eq. (1) where *M*_*1*_ and *M*_*2*_ represent the mass of the hydrogel before and after immersion in ethanol respectively, *p* is the density of absolute ethanol and *V* corresponds to the volume of the hydrogel sample.

(1) $$\text{Porosity}=\left(M_{1}-M_{2}\right)/\mathrm{pV}$$

In addition to porosity, the effect of CaCl_2_ concentration on small molecule diffusion was determined using a sodium fluorescein permeability assay. Hydrogels were crosslinked with 75, 150 and 300 mM CaCl_2_ in 12-well cell culture inserts (0.5 mm pore diameter). After complete gelation, 1 ml of 10 mM sodium fluorescein in dH_2_O was added to the surface of each hydrogel with 1 ml of dH_2_O added into the well below the insert. Absorbance at 490 nm of dH_2_O in the lower well was analysed after 24 and 48 h and data were extrapolated to a standard curve to determine the concentration of sodium fluorescein that had diffused through the hydrogels and into the lower well.

### 2.12 Effect of matrix stiffness on neuronal phenotype

Quantitative RT-PCR was performed as in 2.6 on cell-laden hydrogels reticulated with 75, 150 and 300 mM CaCl_2_. RNA was isolated, converted to cDNA and relative expression of neuron-specific markers MAP2 and synaptophysin was determined using Primerdesign custom primers with GAPDH as a housekeeping gene. Double delta CT was used to calculate expression relative to undifferentiated iPSCs.

### 2.13 Statistical analyses

Quantitative data was tested for significant differences using two-tailed *t*-tests with equal variances assumed. A p value of <0.05 was considered to represent significant differences. All samples were analysed with 3 experimental replicates (n – 3), each containing 3 technical replicates.

## 3 Results

### 3.1 Formation and microstructure of the alginate/collagen hydrogel

**Fig. 2:**
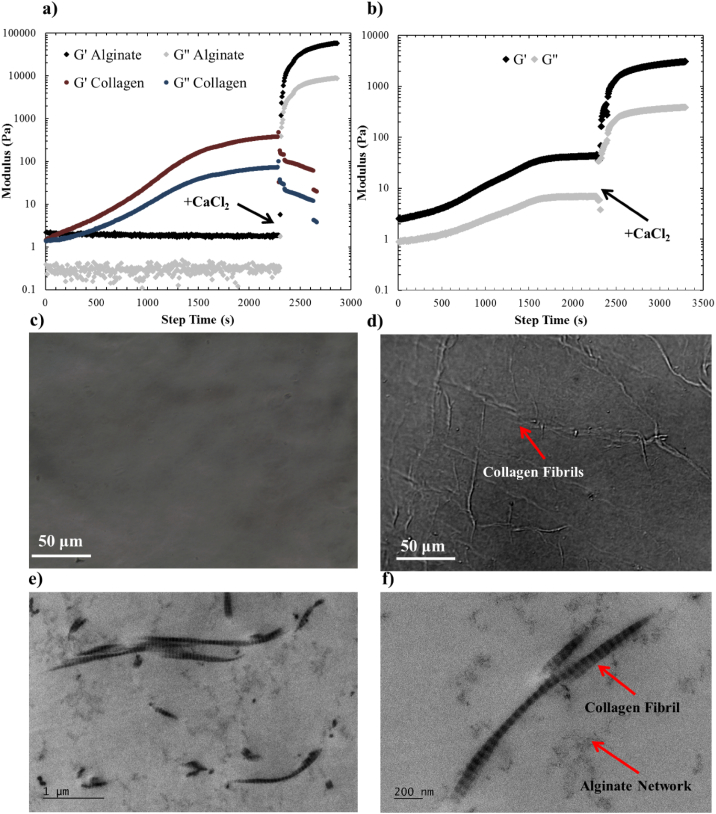
Rheological and structural analyses of alginate/collagen hydrogels. a) Gelation kinetics of collagen and alginate separately via thermal and ionotropic crosslinking, respectively. b) Gelation kinetics of blended alginate/collagen hydrogels. c) Bright field confocal microscopy of a collagen-free alginate hydrogel. d) Bright field confocal microscopy demonstrating the formation and distribution of fibrils within alginate/collagen hydrogels. e–f) High magnification TEM images highlighting the presence of striated collagen fibrils within the alginate matrix. (n = 3 for all data).

Initially, the mechanical properties of the alginate/collagen blended hydrogel were compared with those of alginate and collagen on their own. Time sweeps highlighted distinct differences in gelation mechanics between alginate and collagen when reticulated separately (Fig. 2a). Incubation at 37 °C triggered gelation of collagen; represented by a gradual increase in G′ and G″ prior to the introduction of calcium ions (G′ increased from ~1 Pa to 476 Pa over 40 min). Addition of calcium, however, resulted in a dramatic reduction in both G′ and G″. Alginate, conversely, did not transition from solution to gel until calcium ions were introduced, where a rapid increase in G′ and G″ was observed (G′ increased from ~2 Pa to 10 kPa in 1 min) (Fig. 2a). When both materials were blended and the same reticulation method reproduced, the gelation mechanisms of both materials were observed (Fig. 2b), with a gradual increase in stiffness as the collagenous component gelled followed by rapid ionotropic gelation of alginate with the addition of calcium ions. The resulting hydrogel exhibited a storage modulus of ~2.8 kPa.

Transmission electron microscopy (TEM) also revealed evidence of successful integration of alginate and collagen into a single, heterogeneous matrix via the above gelation mechanism (Fig. 2e–f). Collagen fibrillation was not inhibited within the matrix and alginate polysaccharide networks appeared to co-localise with collagen fibrils. Additionally, bright field confocal images highlighted the spreading of fibrils throughout the hydrogel (Fig. 2d). A lack of similar structures was observed in collagen-free alginate hydrogels (Fig. 2c) indicating that the fibrous network was likely formed by collagen in the blended gels.

### 3.2 The alginate/collagen hydrogel supports cell-matrix adhesions, neurogenesis and neuronal maturation

**Fig. 3:**
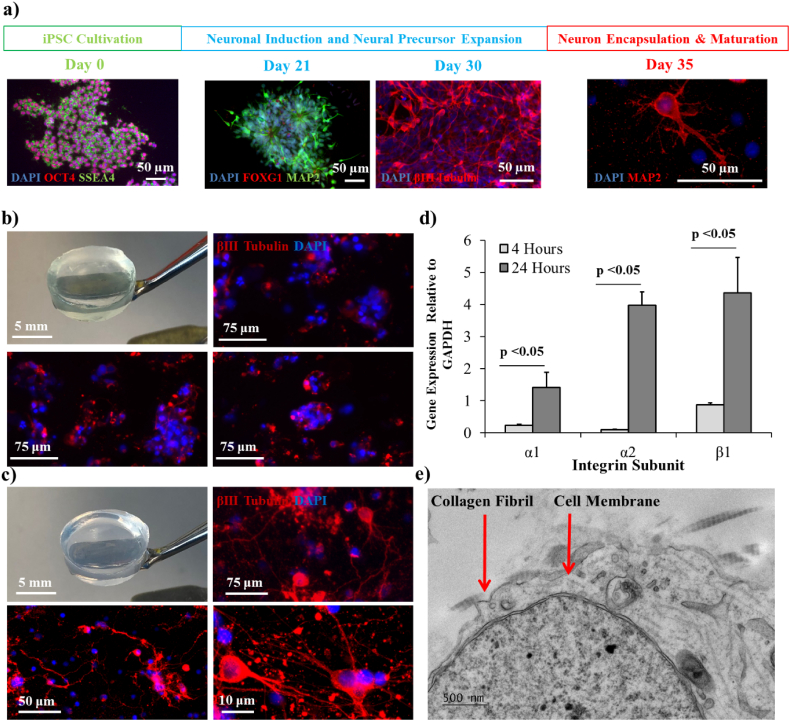
Analysis of the adhesion and morphology of iPSC-derived neurons encapsulated in alginate/collagen hydrogels. a) Timeline of cortical neuron differentiation from human iPSCs with immunofluorescence microscopy staining for stage specific markers highlighting iPSC cultivation (day 0; Oct4 and SSEA4 progenitor markers), neural induction (day 21; FOXG1 and MAP2 neural rosette markers), neural precursor expansion (day 30; βIII tubulin neuronal marker) and encapsulation of differentiated neurons in hydrogels (day 35; MAP2 neuronal marker). b) Immunofluorescence microscopy of the neuronal marker βIII tubulin in neurons encapsulated in collagen-free alginate hydrogels and cultured for 10 days. c) Immunofluorescence microscopy of the neuronal marker βIII tubulin in neurons encapsulated in alginate/collagen blended hydrogels and cultured for 10 days. d) Expression of collagen-binding integrin subunits in iPSC-derived neurons at 4 h and 24 h post-encapsulation in alginate/collagen hydrogels at day 35. e) TEM showing co-localisation of an encapsulated neuron with collagen fibrils within the alginate/collagen hydrogel. (n = 3 for all data, error bars represent standard error of the mean).

Prior to encapsulation, iPSCs were induced down a neuronal lineage and imaged with immunofluorescence microscopy at key time points to determine the success of differentiation prior to cell encapsulation (Fig. 3a). Cells were immunostained over a 35 day period for pluripotency markers, markers of early neuronal differentiation and neural precursor development, and markers of terminally differentiated neurons [39]. After confirmation of a neuronal phenotype, neurons were encapsulated into hydrogel cultures. After 4 h and 24 h of culture in the hydrogels, RT-PCR was used to probe for evidence of encapsulated neurons adhering to the surrounding alginate/collagen hydrogel matrix. Specifically, the expression of collagen binding integrin subunits was measured, as any cell-matrix adhesions would occur via collagen fibrils within the hydrogel structure and not via interactions with alginate, where no cell adhesion motifs are present [27]. All three integrin subunits (α1, α2 and β1) were significantly up-regulated 24 h after encapsulation (Fig. 3d) with α2 showing significantly higher relative expression than α1 (3.98-fold vs 1.41-fold, p = 0.002), suggesting that integrin subunit α2β1 was expressed at higher levels than α1β1 by neurons encapsulated in the hydrogel. Interestingly, TEM of hydrogels seeded with iPSC-derived neurons also indicated the presence of potential cell-matrix interactions with collagen, as adhered neurons were often observed in close proximity to collagen fibrils (Fig. 3e).

The presence of collagen within the surrounding hydrogel matrix also resulted in distinct differences in encapsulated cell morphology when compared with collagen-free alginate hydrogels. Immunofluorescence microscopy demonstrated that in the absence of collagen fibrils, encapsulated neurons adopted a spherical morphology and were unable to project neurites through the alginate hydrogel matrix (Fig. 3b). In contrast, the alginate/collagen hydrogel supported the formation of neural networks (Fig. 3c). Encapsulated neurons projected branched neurites that stained positive for βIII tubulin; a key neural marker involved in generation, guidance and maintenance of neurites during neurogenesis [45]. These data indicate that the alginate/collagen hydrogel provides a suitable environment for the growth and adherence of neurons.

**Fig. 4:**
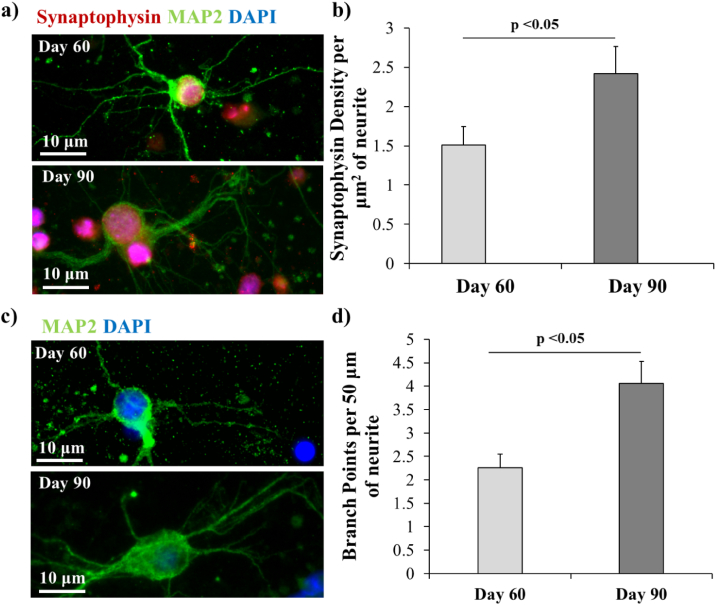
Evaluation of the maturation of neurons encapsulated within the alginate/collagen hydrogel. a) Immunofluorescence microscopy demonstrating synaptophysin location at days 60 and 90 post-neural induction. b) The density of the synaptophysin present in the neurites of encapsulated neurons at day 60 and day 90 post-neural induction was quantified. c) Immunofluorescence microscopy of MAP2 highlighting neurite branching at days 60 and 90 post-induction. d) Average number of branch points per 50 μm of neurite at days 60 and 90 post-induction from the images in (c). (n = 3 for all data, error bars represent standard error of the mean).

**Fig. 5:**
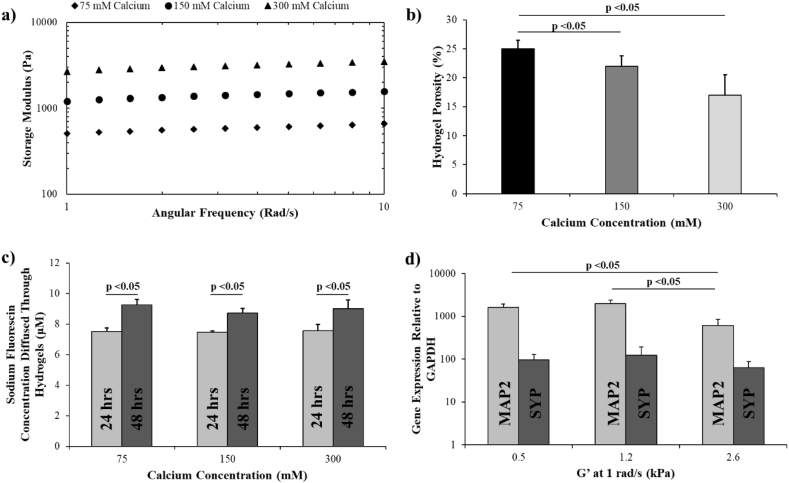
Analysis of the effect of alginate/collagen matrix stiffness on the phenotype of encapsulated neurons. a) Frequency sweeps highlighting the effect of [CaCl_2_] on the storage modulus of alginate/collagen hydrogels. b) Porosity (%) of alginate/collagen hydrogels as a function of [CaCl_2_]. c) The impact of [CaCl_2_] on diffusion of sodium fluorescein through the alginate/collagen hydrogels over 24 h and 48 h. d) The effect of storage modulus of the alginate/collagen hydrogel on the expression of MAP2 and synaptophysin (SYP) in encapsulated iPSC-derived neurons at day 60 post-induction. (n = 3 for all data, error bars represent standard deviation).

At 60 and 90 days post-induction (25 and 55 days after encapsulation in the hydrogel), the iPSC-derived neurons were imaged by immunofluorescence microscopy to determine the intracellular location of synaptophysin, a pre-synaptic vesicle protein that is implicated in the transport of neurotransmitters to the pre-synaptic terminal [46]. After 60 days of neural induction (25 days after encapsulation in the hydrogel), synaptophysin was primarily found in the soma of the neurons with very little observed in the neurites (Fig. 4a). Culturing the neurons for a further 30 days within the hydrogels (90 days post-induction, 55 days post encapsulation) resulted in a significant increase in the amount of synaptophysin observed within the neurites. Quantification of the fluorescent density of synaptophysin revealed a significant increase at day 90 when compared with day 60 (Fig. 5b; 2.4 vs 1.5, p = 0.03). Additionally, the neurons exhibited a change in morphology over the 90 day culture period with formation of neurites containing significantly more branch points resulting in more complex neural networks (Fig. 4c–d; 2 vs 4, p = 0.001). These data indicate that encapsulation of the iPSC-derived neurons in the alginate/collagen hydrogel allowed for sustained neuronal maturation.

### 3.3 The mechanical and structural properties of the alginate/collagen hydrogel influence neuronal maturation

Rheological analysis of the alginate/collagen hydrogels crosslinked with increasing concentrations of CaCl_2_ revealed a positive relationship between CaCl_2_ concentration and storage modulus of the resulting gel (Fig. 5a). Hydrogels crosslinked with 75 mM CaCl_2_ exhibited a G′ of ~0.5 kPa at a frequency of 1 rad/s. At the same frequency, moduli of 1.2 kPa and 2.6 kPa were observed with 150 mM and 300 mM CaCl_2_, respectively. Additionally, hydrogel porosity decreased significantly as a function of increased CaCl_2_ concentration. Alginate/collagen hydrogels exhibited a mean porosity of 25%, 20% and 17% when crosslinked with 75 mM, 150 mM and 300 mM CaCl_2_ respectively (Fig. 5b). Small molecule diffusion was, however, unaffected by changes in porosity as demonstrated by equal diffusion of sodium fluorescein though the alginate/collagen hydrogels crosslinked with all 3 concentrations of CaCl_2_ (Fig. 5c). After 24 h, there was no difference in the amount of sodium fluorescein that diffused through each hydrogel (p > 0.05). After 48 h, despite a significant increase in the overall amount of sodium fluorescein that had diffused through the hydrogel samples, there was again no difference between the hydrogels in the amount that had diffused across. This suggests that small molecule diffusion is not inhibited by the variable crosslinking in the hydrogels and occurs at a similar rate despite the changes in bulk porosity.

Neurons cultured for 14 days in an alginate/collagen hydrogel crosslinked with a 75 mM CaCl_2_ exhibited no significant differences in the expression of either MAP2, a key marker of neuronal differentiation and maturation [47], or synaptophysin when compared to neurons cultured in a hydrogel crosslinked with a 150 mM CaCl_2_ (Fig. 5d). However, when CaCl_2_ concentration was increased to 300 mM, there was a significant decrease in expression of MAP2 in the neurons, and a reduction, albeit not significant, of synaptophysin. Together, these data indicate that the mechanical and structural properties of the alginate/collagen hydrogel can be easily manipulated and that such changes can impact the maturation of the neurons.

## 4 Discussion

This study demonstrates a simple method for generating a customisable hydrogel platform for the 3D culture of human iPSC-derived neurons by combining two naturally available polymers, namely alginate and collagen. Alginate is an attractive material for such applications due to structural similarities with hyaluronic acid, although the requirement of chemical modification to stimulate cell-matrix adhesions impairs its use [23]. Here we show that blending collagen with alginate and fully controlling the gelation mechanism yields a mechanically tuneable hydrogel. The resulting hydrogel matrix is a heterogeneous network of crosslinked alginate and collagen fibrils that facilitate cell attachment, neuronal maturation and mechanotransductive responses. Moreover, the hydrogel formation can be initiated under physiological conditions, thus aiding long term cell viability.

Rheological studies of alginate/collagen blends demonstrated gelation of both materials was not inhibited when they were physically mixed together (Fig. 2). An initial gradual increase in modulus prior to addition of calcium ions can likely be attributed to the ordering of collagen into a hydrogel network as samples were incubated at 37 °C throughout the gelation study. At this temperature, a solution of collagen molecules will pack together to form a tight structure of crosslinked collagen fibrils, resulting in a gradual increase in matrix stiffness and formation of a hydrogel [48]. This mechanism was observed in gelation studies of collagen alone (Fig. 2a) and the gelation profile of the collagen/alginate blend closely matched that of collagen prior to addition of calcium (Fig. 2b). Additionally, as the collagen network formed it is likely that the alginate within the blend was still in the liquid phase, as analysis of alginate alone indicated that gelation was not initiated until calcium ions were introduced to the system. Addition of calcium into the system after 40 min resulted in a rapid increase in modulus due to ionotropic gelation of alginate [49], yielding a single bulk gel containing both raw materials (Fig. 2b).

Formation of collagen fibrils within the blended hydrogel (Fig. 2d–f) could potentially be due to alginate remaining in a liquid phase during the gelation of collagen. A previous study by Gillette et al. [50] demonstrated that when alginate was present as a crosslinked hydrogel, collagen fibrillation was physically inhibited. When alginate was in the liquid phase, however, collagen fibrils penetrated and spread through the alginate within 40 min. The resulting structure was then crosslinked to create an alginate hydrogel penetrated by collagen fibrils. During gelation of the alginate/collagen blend for cell culture applications, calcium ions were not introduced into the system until after a 4 h incubation at 37 °C allowing time for the collagen fibrils to form a network that spread through the alginate solution prior to gelation, resulting in an alginate hydrogel penetrated by collagen fibrils.

Neurons encapsulated within the blended hydrogel appeared to interact and bind to collagen fibrils present within the hydrogel matrix (Fig. 3e). This is supported by a significant upregulation in collagen binding integrins α1β1 and α2β1 at 24 h post-encapsulation (Fig. 3d), suggesting that the neurons can locate and adhere to collagen fibrils that are spread throughout the alginate polysaccharide network. Additionally, neuron-collagen interactions are potentially aided by the uniform distribution of fibrils through the matrix (Fig. 2d) which could facilitate availability of a high number of integrin binding sites. Integrin α1β1 and α2β1 compete for the same binding site in collagen [51], thus the higher expression of the α2 subunit suggests a potential preference for α2β1 mediated collagen adhesion in the encapsulated neurons.

Adherence of encapsulated neurons to the hydrogel network facilitated the formation of neurites and generation of a neural network (Fig. 3, Fig. 4). In the absence of collagen fibrils and, thus, a lack of cell adhesion domains within the alginate hydrogel structure [52], encapsulated neurons adopted a spherical morphology and were unable to form neurites (Fig. 3b). Formation of a complex network of neurites by neurons within the alginate/collagen blend was, therefore, most likely directly due to the penetration of collagen fibrils throughout the hydrogel microstructure allowing for the adhesion of neurons to the surrounding matrix.

Formation of a 3D neural network also triggered a key mechanism of neuronal maturation, namely translocation of synaptophysin from the cell soma down the neurites (Fig. 4a–b) [53,54]. Synaptophysin is involved with neurotransmission between functional neurons [46]. Overexpression of synaptophysin has been shown to enhance neurotransmission and the protein is involved in regulating endocytosis at presynaptic terminals and sustaining electrical activity between neurons [46,55]. The presence of synaptophysin within neurites has, therefore, become a marker for maturation into neurons capable of transmitting electrical signals [56,57]. The presence of synaptophysin within the neurites of neurons encapsulated within alginate/collagen hydrogels at day 90 post-differentiation indicates maturation into functional neurons; a process that can often be limited in hydrogel culture [58,59]. This is further supported by morphological changes observed in encapsulated neurons over the 90 day culture period (Fig. 4c–d). Neuronal processes became more branched, with neurites at day 90 post-induction having significantly more branch points than at day 60 post-induction. Formation of more branched neural processes is another key marker of neuronal maturation [60] and, paired with synaptophysin data, suggests that encapsulated neurons are actively developing a functional neuronal phenotype within the hydrogel.

Differential expression of MAP2 in response to changes in mechanical and structural properties (Fig. 5) also suggests that encapsulated neurons receive physical cues from the bulk hydrogel structure, and not just the collagen fibrils. Increases in stiffness with calcium dosing are a result of further crosslinking of alginate as collagen is not natively capable of cationic crosslinking [48,49]. At 2.6 kPa, encapsulated neurons were exposed to a matrix stiffness that is at least twofold greater than the higher estimates commonly reported for human brain [3]. This environment triggered significant downregulation of MAP2, a marker for neuronal maturation and neural network formation and stabilisation [61]. Previous studies have demonstrated that soft substrates enhance neurogenesis and neuronal maturation by initiating increased MAP2 expression and inducing formation of a neural network at a greater rate. Stiffer substrates are, conversely, often reported to inhibit this process [62,63]. Synaptophysin expression was not significantly affected by changes in matrix stiffness, consistent with recent analyses demonstrating that unlike neurogenesis, stiffer substrates may not negatively impact neurotransmission in encapsulated neurons [64,65].

Another possible explanation for the changes in MAP2 expression relates to the microarchitecture of the hydrogels. The alginate hydrogels exhibiting a G′ of 2.6 kPa were significantly less porous than the softer counterparts (Fig. 5b). Changes in porosity within a hydrogel can also play a significant role in regulating cell phenotype. Altered hydrogel microarchitecture can impact on the proliferation and migration of encapsulated cells with increased porosity often inversely affecting proliferative and migratory behaviour [66]. Due to its role in neuronal migration it is, therefore, worth considering that the decrease in hydrogel porosity also contributed to a reduction in MAP2 expression [67]. Additionally, changes in hydrogel porosity can impact on diffusion of nutrients and other essential small molecules from the cell culture media. However, porosity did not negatively impact on small molecule diffusion within the hydrogels as assessed with sodium fluorescein (Fig. 5c).

## 5 Conclusions

Rheological, TEM and confocal microscopy analyses of alginate/collagen hydrogels indicate that both materials were successfully integrated into a single blended hydrogel structure. The gelation of both materials was not inhibited and collagen fibrils successfully formed within the hydrogel matrix. Encapsulated human iPSC-derived neurons successfully adhered to the hydrogel matrix as evidenced by co-localisation to collagen fibrils and subsequent upregulation of collagen binding integrins. This resulted in formation of a 3D network of neurites and maturation into branched neurons expressing synaptophysin, a protein involved in neurotransmission, along the neurites. Additionally, tuning the mechanical and structural properties of the hydrogel by simple modulation of ionic crosslinker concentration influenced cell phenotype and allowed for the optimisation of neuron-specific gene expression within the hydrogel. Alginate/collagen hydrogels could, therefore, be applied as bespoke substrates for studying neuronal responses to different mechanical and structural environments, directing 3D neurogenesis and studying neuronal behaviour in 3D cell culture models.

## Data Availability

The authors declare that all data supporting the findings of this study are available within the paper. Source data for the figures in this study are available from the authors upon reasonable request.
